# Partisan Differences in Twitter Language Among US Legislators During the COVID-19 Pandemic: Cross-sectional Study

**DOI:** 10.2196/27300

**Published:** 2021-06-03

**Authors:** Sharath Chandra Guntuku, Jonathan Purtle, Zachary F Meisel, Raina M Merchant, Anish Agarwal

**Affiliations:** 1 Department of Computer and Information Science University of Pennsylvania Philadelphia, PA United States; 2 Penn Medicine Center for Digital Health University of Pennsylvania Philadelphia, PA United States; 3 Leonard Davis Institute of Health Economics University of Pennsylvania Philadelphia, PA United States; 4 Department of Health Management & Policy Drexel University Dornsife School of Public Health Philadelphia, PA United States; 5 Department of Emergency Medicine University of Pennsylvania Philadelphia, PA United States; 6 Center for Emergency Care Research and Policy University of Pennsylvania Philadelphia, PA United States

**Keywords:** Twitter, COVID-19, digital health, US legislators, natural language processing, policy makers, social media, policy, politics, language, cross-sectional, content, sentiment, infodemiology, infoveillance

## Abstract

**Background:**

As policy makers continue to shape the national and local responses to the COVID-19 pandemic, the information they choose to share and how they frame their content provide key insights into the public and health care systems.

**Objective:**

We examined the language used by the members of the US House and Senate during the first 10 months of the COVID-19 pandemic and measured content and sentiment based on the tweets that they shared.

**Methods:**

We used Quorum (Quorum Analytics Inc) to access more than 300,000 tweets posted by US legislators from January 1 to October 10, 2020. We used differential language analyses to compare the content and sentiment of tweets posted by legislators based on their party affiliation.

**Results:**

We found that health care–related themes in Democratic legislators’ tweets focused on racial disparities in care (odds ratio [OR] 2.24, 95% CI 2.22-2.27; *P*<.001), health care and insurance (OR 1.74, 95% CI 1.7-1.77; *P*<.001), COVID-19 testing (OR 1.15, 95% CI 1.12-1.19; *P*<.001), and public health guidelines (OR 1.25, 95% CI 1.22-1.29; *P*<.001). The dominant themes in the Republican legislators’ discourse included vaccine development (OR 1.51, 95% CI 1.47-1.55; *P*<.001) and hospital resources and equipment (OR 1.22, 95% CI 1.18-1.25). Nonhealth care–related topics associated with a Democratic affiliation included protections for essential workers (OR 1.55, 95% CI 1.52-1.59), the 2020 election and voting (OR 1.31, 95% CI 1.27-1.35), unemployment and housing (OR 1.27, 95% CI 1.24-1.31), crime and racism (OR 1.22, 95% CI 1.18-1.26), public town halls (OR 1.2, 95% CI 1.16-1.23), the Trump Administration (OR 1.22, 95% CI 1.19-1.26), immigration (OR 1.16, 95% CI 1.12-1.19), and the loss of life (OR 1.38, 95% CI 1.35-1.42). The themes associated with the Republican affiliation included China (OR 1.89, 95% CI 1.85-1.92), small business assistance (OR 1.27, 95% CI 1.23-1.3), congressional relief bills (OR 1.23, 95% CI 1.2-1.27), press briefings (OR 1.22, 95% CI 1.19-1.26), and economic recovery (OR 1.2, 95% CI 1.16-1.23).

**Conclusions:**

Divergent language use on social media corresponds to the partisan divide in the first several months of the course of the COVID-19 public health crisis.

## Introduction

The novel COVID-19 pandemic continues to surge throughout the world. The United States’ federal and state policy responses continue to shift and vary throughout the stages of the pandemic [1]. Notable divisions related to public health measures and frameworks for closing and reopening local economies have proliferated [2]. A unique aspect of the COVID-19 pandemic is the role that social media plays in housing, disseminating, and amplifying information and opinions [3,4]. US legislators have also taken to social media to connect with their constituents, comment on the pandemic, and provide information across a spectrum of pandemic-related content to individuals.

Understanding what content US legislators are sharing through social media posts (eg, Twitter) and how they are relaying COVID-19–related information is important, as these issues guide public knowledge and public opinion and inform policy change. By using social media data, prior studies have identified growing partisan differences among Republican and Democrat legislators as the pandemic has progressed [5]. It has also been found that tweets about specific topics (eg, social distancing) from legislators are often associated with the time when policies are put into action, and the effect of such tweets are larger in democratic counties [6].

The objective of this study was to analyze the language in posts on Twitter—a leading social media platform—that were posted by US legislators over the course of the pandemic to identify potential health care–related themes in COVID-19–related posts and to analyze the associated sentiment within tweet language across partisans.

## Methods

### Data

We identified state legislators’ Twitter posts that were related to COVID-19 and posted from January 1 to October 10, 2020, by using Quorum (Quorum Analytics Inc) [7], a software platform that collects policy-related documents, including social media posts from politicians that were posted during their time in office. This study was considered exempt from review by the University of Pennsylvania Institutional Review Board, as it involves the analysis of public-facing data.

### Language Feature Extraction

We extracted the relative frequency of single words and phrases from tweets by using the Differential Language Analysis ToolKit package [8] and created two sets of features—(1) an open vocabulary that was defined by using latent Dirichlet allocation [9], an unsupervised clustering algorithm, to create 50 data-driven word clusters (topics) and (2) sentiment, which was measured by using the National Research Council (NRC) Canada lexicon [10], a data-driven dictionary containing words associated with positive and negative valence. The NRC lexicon was developed by using a corpus of 77,500 positive and negative tweets, and consists of 54,129 weighted unigrams and 316,531 bigrams in which the weight corresponds to the degree of association between a token and sentiment [10].

### Statistical Analyses

To distinguish linguistic differences across political parties (coded as a dichotomous outcome), each feature set was input in a logistic regression model, and those that were significantly different according to a cutoff Benjamini-Hochberg–corrected *P* value of <.001 were reported [11]. Two authors independently evaluated each topic for thematic meanings by reviewing the top 10 posts per topic and coded them into health care–related and nonhealth care–related themes.

Data on changes in the prevalence and sentiment of topics that were significantly associated with either party and occurred over time were obtained by calculating the mean scores across all posts per week, stratified by party, and visualized via locally estimated scatterplot smoothing [12].

## Results

### US Legislators’ Tweets

**Figure 1:**
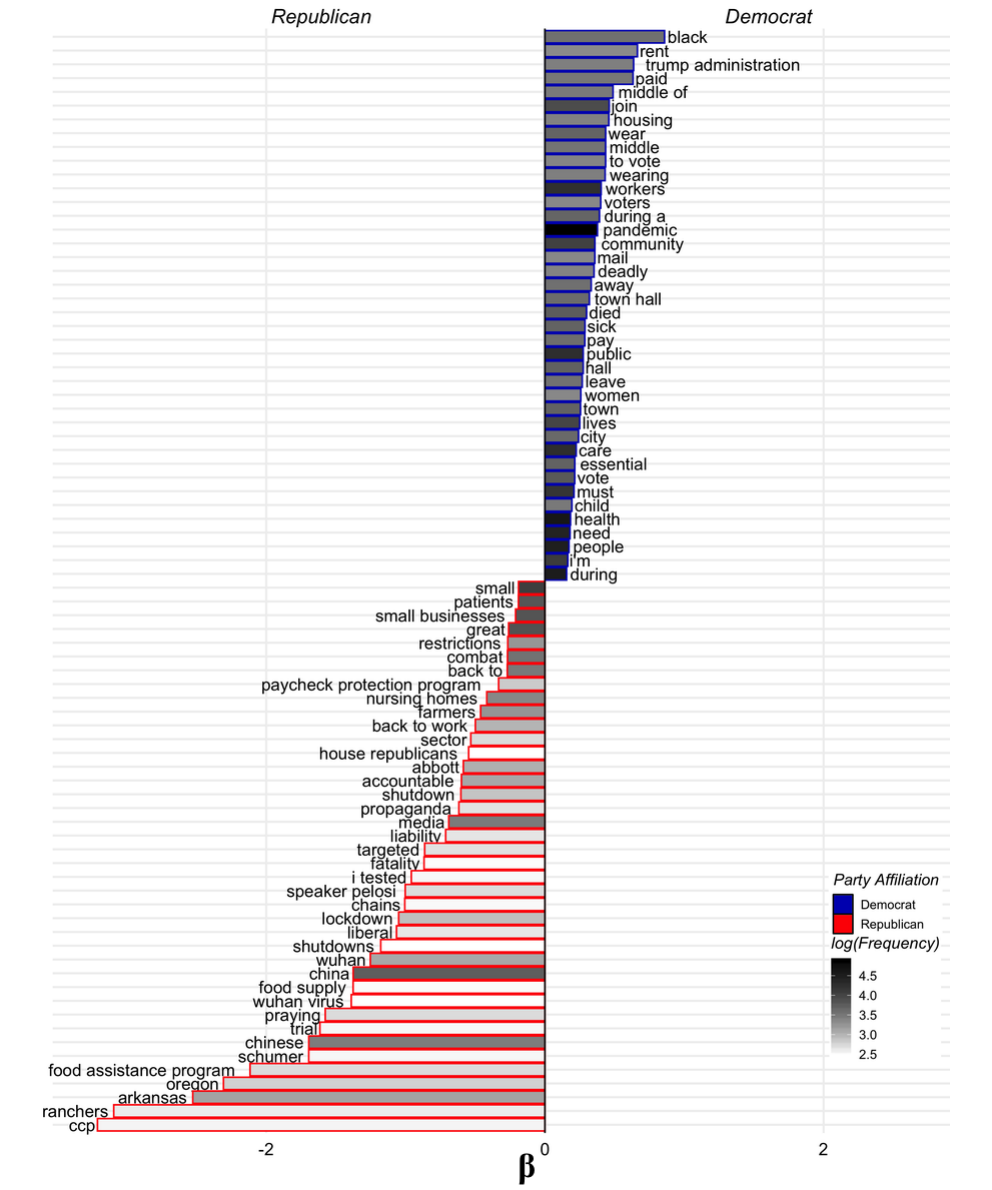
Words and phrases that were significantly associated with tweets from Democratic legislators (blue) and Republican legislators (red). Bar length indicates effect size and shade indicates relative word frequency (*P*<.001; Benjamini-Hochberg p-correction). CCP: Chinese Communist Party.

We identified 309,438 COVID-19–related tweets from the 4224 unique accounts of US legislators. The descriptive statistics of the data set are in Table 1. The number of tweets per legislator over the selected time period is shown in Multimedia Appendix 1. Tweet language that correlated with US legislature party affiliation is displayed in Figure 1. Of the statistically significant topics, we identified 7 health care–related themes and 14 nonhealth care–related themes associated with the two major party affiliations.

**Table 1:** Descriptive statistics of the data collected from Quorum from January 1 to October 10, 2020.

Category		Value, n (%)
**Number of tweets**		
	All legislators	309,438 (100)
	Republican legislators	88,146 (28.4)
	Democrat legislators	221,292 (71.5)
**Number of retweets**		
	Republican retweets	38,120 (12.3)
	Democrat retweets	96,469 (31.1)
**Number of individual accounts**		
	All legislators	4224 (100)
	Democrat legislators	2432 (57.6)
	Republican legislators	1792 (42.4)

### Thematic Differences by Party Affiliation

Health care–related themes (Table 2) associated with a Democratic party affiliation included the following: racial disparities in care (odds ratio [OR] 2.24, 95% CI 2.22-2.27), health care and insurance (OR 1.74, 95% CI 1.70-1.77), public health guidelines (OR 1.25, 95% CI 1.22-1.29), and COVID-19 testing (OR, 1.15, 95% CI 1.12-1.19). Health care–related themes associated with a Republican party affiliation included the following: vaccine development (OR 1.51, 95% CI 1.47-1.55) and hospital resources and equipment (OR 1.22, 95% CI 1.18-1.25).

Nonhealth care–related topics were also identified across parities. The themes associated with a Democratic affiliation included the following: protections for essential workers, the 2020 election and voting, unemployment and housing, crime and racism, public town halls, the Trump Administration, immigration, and the loss of life. The themes associated with a Republican affiliation included the following: China, congressional relief bills, small business assistance, press briefings, and economic recovery (Table 3). The prevalence of the themes over time stratified by affiliation is shown in Multimedia Appendices 2 and 3. The set of topics that did not significantly correlate with affiliation are shown in Multimedia Appendix 4.

**Table 2:** Health care–related topics that are more likely to be posted by Democrat legislators and Republican legislators. Effect size is shown by using odds ratios (ORs) along with 95% CIs. Only significant topics after Benjamini-Hochberg p-correction (*P*<.001) are shown.

Affiliation and topic theme		Top words	ORs (95% CI)	Example tweets
**Democrat**				
	Racial disparities	“communities,” “black,” “color,” “racial,” “disparities,” “disproportionately,” “impact,” “hit,” “women,” and “latino”	2.24 (2.22-2.27)	“COVID-19 is disproportionately harming communities of color and exposing generations of systemic racism. We need to collect racial and ethnic data for coronavirus testing and treatment so we can address these health disparities and begin rectifying decades of injustice.”
	Health care and insurance	“healthcare,” “americans,” “access,” “insurance,” “coverage,” “affordable,” “millions,” “medicaid,” and “court”	1.74 (1.70-1.77)	“The #MedicareCrisisProgram would: Expand Medicare to the recently unemployed & cap out-of-pocket costs Expand Medicaid to cover even more people Ensure no out-of-pocket costs for COVID-19 testing/treatment for everyone”
	Public health guidelines	“mask,” “social,” “distancing,” “wear,” “spread,” “hands,” “stay,” “home,” “wash,” “practice,” and “stop”	1.25 (1.22-1.29)	“The pandemic is nowhere near over. Continue practicing social distancing & WEAR A MASK”
	COVID-19 testing	“testing,” “free,” “county,” “sites,” “appointment,” “center,” “residents,” “open,” “city,” “visit,” and “symptoms”	1.15 (1.12-1.19)	“New COVID-19 mobile testing site opens. Scheduled Locations for Free Drive-Through COVID-19 Testing” [retweet]
**Republican**				
	Vaccine development	“vaccine,” “research,” “drug,” “production,” “effective,” “vaccines,” “treatments,” “development,” and “defense”	1.51 (1.47-1.55)	“Three Coronavirus Vaccine Developers Report Promising Initial Results” [retweet]
	Hospital resources and equipment	“medical,” “patients,” “hospitals,” “masks,” “equipment,” “supplies,” “ppe,”^a^ “donate,” “blood,” “needed,” and “plasma”	1.22 (1.18-1.25)	“Kansans everywhere are stepping up to fight the #Coronavirus.…,which manufactures aircraft parts in…, is using their 3D printing capabilities to work with local area hospitals on prototypes of N-95 surgical masks and protective face shields.”

^a^PPE: personal protective equipment.

**Table 3:** Nonhealth care–related topics that are more likely to be posted by Democratic legislators and Republican legislators. Effect size is shown by using odds ratios (ORs) along with 95% CIs. Only significant topics after Benjamini-Hochberg p-correction (*P*<.001) are shown.

Affiliation and topic theme				Top words		OR (95% CI)		Example tweets
**Democrat**								
	Protections for essential workforce			“workers,” “essential,” “leave,” “sick,” “pay,” “employees,” “safety,” “job,” “frontline,” “grocery,” “protections,” and “deserve”		1.55 (1.52-1.59)		“Essential workers--like farmworkers, first responders, health care workers, and grocery store workers--deserve hazard pay from their government or their company for their service during the #coronavirus pandemic.”
	2020 election and voting			“vote,” “mail,” “voters,” “ballot,” “elections,” “absentee,” “november,” “census,” “primary,” “voter,” “ballots,” and “2020”		1.31 (1.27-1.35)		“To make sure this virus doesn't keep people from the ballot box, states and localities should bring the ballot box to them through expanded vote-by-mail and no-fault absentee voting. #SAFEDemocracy”
	Unemployment and housing assistance			“unemployment,” “assistance,” “benefits,” “rent,” “program,” “housing,” “insurance,” “eviction,” “eligible,” “lost,” and “claims”		1.27 (1.24-1.31)		“Wisconsin residents who have exhausted their regular unemployment insurance (UI) benefits may now apply for Pandemic Emergency Unemployment Compensation (PEUC).”
	Crime and racism			“violence,” “police,” “domestic,” “racism,” “asian,” “stand,” “hate,” “gun,” “survivors,” “protests,” “discrimination,” and “victims”		1.22 (1.18-1.26)		“As coronavirus fears have intensified, incidents of violence & discrimination against Chinese Americans have increased. Joined hundreds of San Franciscans marching in Chinatown today to protest prejudice and racial profiling. #TogetherWeStand #StandWithChinatown”
	Public town halls			“join,” “hall,” “town,” “live,” “questions,” “discuss,” “tomorrow,” “tune,” “facebook,” “virtual,” “i'll,” “telephone,” “tonight”		1.2 (1.16-1.23)		“Tomorrow evening, at 7:30 pm EST [Eastern Standard Time], I am hosting another Coronavirus Telephone Town Hall.”
	**Trump Administration**							
		Word set 1	“trump,” “national,” “service,” “global,” “administration,” “postal,” “guard,” “security,” “decision,” “usps,”^a^ and “members”		1.22 (1.19-1.26)		“Donald Trump and Mike Pence's handling of COVID-19 is the greatest failure of any American presidency.…#Debate2020 #TrumpFailure #IwillVote”	
		Word set 2	“trump,” “president,” “white,” “house,” “trump's,” “donald,” “don't,” “biden,” “he's,” “joe,” “administration,” “campaign,” “pence,” “force,” and “rally”		1.17 (1.13-1.2)		“Democratic presidential candidate Joe Biden criticized President Donald Trump's 'callousness' in handling the coronavirus pandemic” [retweet]	
	Immigration			“letter,” “release,” “colleagues,” “risk,” “prisons,” “urging,” “ice,”^b^ “immigration,” “inmates,” “detention,” and “vulnerable”		1.16 (1.12-1.19)		“ICE must suspend immigration enforcement during the #COVID19 pandemic.”
	**Loss of life**							
		Word set 1	“trump,” “americans,” “lives,” “president,” “american,” “died,” “100,” “million,” “deaths,” “200,” “states,” “lost,” “dead,” and “leadership”		1.38 (1.35-1.42)		“In trump's catastrophic zeal to lie about the coronavirus threat, innumerable Americans' lives are in danger.”	
		Word set 2	“family,” “lost,” “friends,” “loved,” “died,” “life,” “heart,” “loss,” “years,” “god,” “prayers,” “remember,” “thoughts,” and “honor”		1.13 (1.09-1.16)		“Within a few short days, 200,000 mothers fathers daughters sons children parents lovers wives husbands friends grandparents aunts uncles cousins DEAD of coronavirus. We dare not get numb. That equates to every person...EVERY single person in my community.”	
**Republican**								
	China			“china,” “world,” “chinese,” “accountable,” “communist,” “global,” “hold,” “party,” “wuhan,” “china's,” “outbreak,” “government,” “travel,” “held,” and “organization”		1.89 (1.85-1.92)		“Pompeo slams communist China for lying about Wuhan coronavirus” and “The Wuhan virus is #MadeInChina.” [retweet]
	Small business assistance			“small,” “businesses,” “program,” “relief,” “loans,” “impacted,” “apply,” “owners,” “economic,” “grants,” “assistance,” “grant,” and “disaster”		1.27 (1.23-1.3)		“Small businesses impacted by the #coronavirus can apply for a low-interest SBA [Small Business Association] disaster loan here”
	**Government relief funds**							
		Word set 1	“funding,” “federal,” “local,” “state,” “million,” “act,” “support,” “relief,” “communities,” “cares,” “resources,” “governments,” “provide,” and “emergency”		1.22 (1.18-1.25)		“Democrats Filibuster Covid Relief” [retweet]	
		Word set 2	“relief,” “senate,” “bill,” “republicans,” “democrats,” “house,” “americans,” “mcconnell,” “pass,” “american,” “pelosi,” “congress,” “package,” “politics,” and “gop”^c^		1.23 (1.2-1.27)		“In the CARES [Coronavirus Aid, Relief, and Economic Security] Act,…received money to be disbursed ASAP [as soon as possible] to help local communities fight coronavirus. Grants were disbursed through specific programs, including Community Development Block Grants-flexible funding to states and local governments-Steubenville awarded $365,667!”	
	**Press briefings**							
		Word set 1	“governor,” “update,” “press,” “north,” “conference,” “gov,” “watch,” “live,” “carolina,” “#ncpol,”^d^ “michigan,” “nc,”^e^ “south,” “state's,” and “briefing”		1.22 (1.19-1.26)		“What is Montana doing in response to coronavirus?” COVID-19 Montana state response update.”	
		Word set 2	“hearing,” “response,” “discuss,” “committee,” “watch,” “force,” “joined,” “task,” “meeting,” “dr,”^f^ “morning,” “impact,” “talk,” “yesterday,” and “hear”		1.14 (1.1-1.17)		“#ICYMI [in case you missed it] I discussed the #Coronavirus in depth on the latest episode of #TheBreakDown.”	
	State politics			“georgia,” “court,” “power,” “supreme,” “restrictions,” “republican,” “politics,” “tennessee,” “decision,” “law,” “abortion,” and “wisconsin”		1.16 (1.12-1.19)		“6th Circuit unanimously rules DWP (drunk-with-power) Beshear ban on church services unconstitutional! Kentucky coronavirus”
	**Economic recovery and news**							
		Word set 1	“economy,” “economic,” “back,” “jobs,” “recovery,” “plan,” “recover,” “america,” “future,” “nation,” “forward,” “industry,” “climate,” “energy,” and “safely”		1.19 (1.15-1.22)		“As a nation, we will defeat the coronavirus and rebuild the greatest economy. #CommitmentToAmerica”	
		Word set 2	“news,” “times,” “york,” “data,” “good,” “washington,” “thread,” “study,” “post,” “breaking,” “shows,” “found,” and “months”		1.20 (1.16-1.23)		“Germany's R0 coronavirus experiment: Berlin tries to manage a variable no one can measure accurately”	

^a^USPS: US Postal Service.

^b^ICE: Immigration and Customs Enforcement.

^c^GOP: Grand Old Party.

^d^NCPOL: North Carolina Political News.

^e^NC: North Carolina.

^f^DR: doctor.

### Sentiment Differences by Party Affiliation

**Figure 2:**
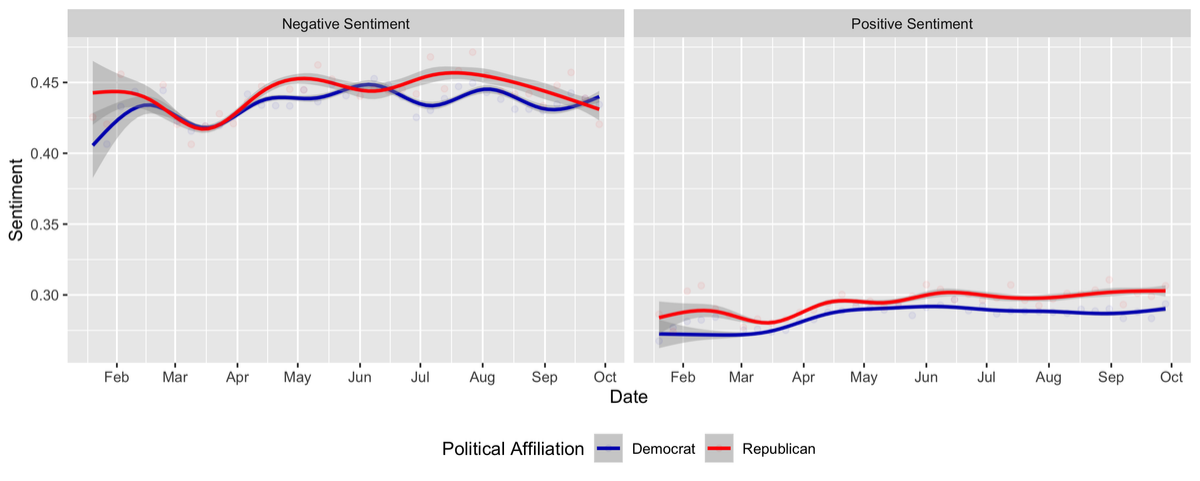
Sentiment analysis of US legislators’ language on Twitter across party affiliations.

We performed an analysis of sentiment for the language used in tweets and found that overall, Republican-affiliated tweets used more positive sentiment, which increased over time. The variation in overall sentiment is shown in Figure 2. Negative sentiment was associated with content from both parties across the following themes: health care and insurance, COVID-19 testing, and racial disparities. Positive sentiment was associated with content within the theme of government public health expertise. Sentiment within themes over time and across parities is identified in Multimedia Appendices 5 and 6.

## Discussion

By using machine learning techniques, we investigated narrative content in over 300,000 twitter posts from US legislators over the course of the COVID-19 pandemic to date. Investigating the language within posts on social media platforms has become more common and has been specifically used to study aspects of health and health care. This study is among the first to analyze US legislators’ Twitter-based language to identify the COVID-19–related themes that policy makers are discussing on Twitter with a specific focus on health care–related topics. Additionally, this study deployed advanced language assessments that use machine learning to analyze how legislators are talking about these themes by conducting sentiment analyses throughout the phases of the pandemic.

We noted key differences across the two major US political parties. Health care–related themes that correlated with a Democratic party affiliation focused on the health care access and disparities across race. The themes that correlated with a Republican party affiliation focused on initial and persistent vaccine progress, access to equipment (eg, personal protective equipment), and government expertise. Furthermore, in the language analysis, we identified that across content posted by Republican legislators, there was considerably more content about the pandemic and approaches for managing the pandemic across health care topics. Language analysis was also used to detect thematic differences in narrative content within Twitter posts across the two major political parties. In this study, our results indicated that legislators with a Democratic party affiliation focused their COVID-19 content more toward social services and racial disparities. Content from Republican-affiliated legislators focused thematically on government relief and economic aid. This finding is consistent with surveys of elected officials and the general public, which suggests that awareness and concern about health disparities among Democrats are greater than those among Republicans [13,14].

There are limitations to this study, including the fact that content was collected from publicly available Twitter posts; thus, legislators who do not post content were not included. If a legislator did not have a party affiliation (as noted by the Quorum database), we could not include them in this analysis. We also did not control for demographic or health access data, as our analysis was performed on the language of individual legislators. Further, a topic’s significant association with a particular affiliation does not imply that other party legislators did not tweet about it; it only indicates the relative frequency of tweets containing the words that were associated with each topic.

This study highlights the ability to understand how legislators use social media (eg, Twitter); what information they choose to share; and how they frame their content, which was determined through sentiment analysis [15]. These are key insights that will remain important to the public and health care systems as policy makers continue to shape the national and local responses to the pandemic [16].

## Appendix

Multimedia Appendix 1 Mean number of COVID-19–related tweets per legislator by party affiliation. The tweets in our data set were posted from January 1 to October 10, 2020.

Multimedia Appendix 2 Prevalence of health care–related topics over time by party affiliation.

Multimedia Appendix 3 Prevalence of nonhealth care–related topics over time by party affiliation.

Multimedia Appendix 4 Topics that were associated with Democrat (top) and Republican (bottom) legislators' Twitter language but did not pass the statistical significance threshold.

Multimedia Appendix 5 Sentiment of COVID-19–related legislator tweets on health care topics.

Multimedia Appendix 6 Sentiment of COVID-19–related legislator tweets on nonhealth care topics.

## Abbreviations

HCV: hepatitis C virus
NRC: National Research Council
OR: odds ratio
