# Supplementary figures and images for: Partisan Differences in Twitter Language Among US Legislators During the COVID-19 Pandemic: Cross-sectional Study
Source: J Med Internet Res. 2021 Jun 3;23(6):e27300. doi: 10.2196/27300 (PMC8176946; doi:10.2196/27300)

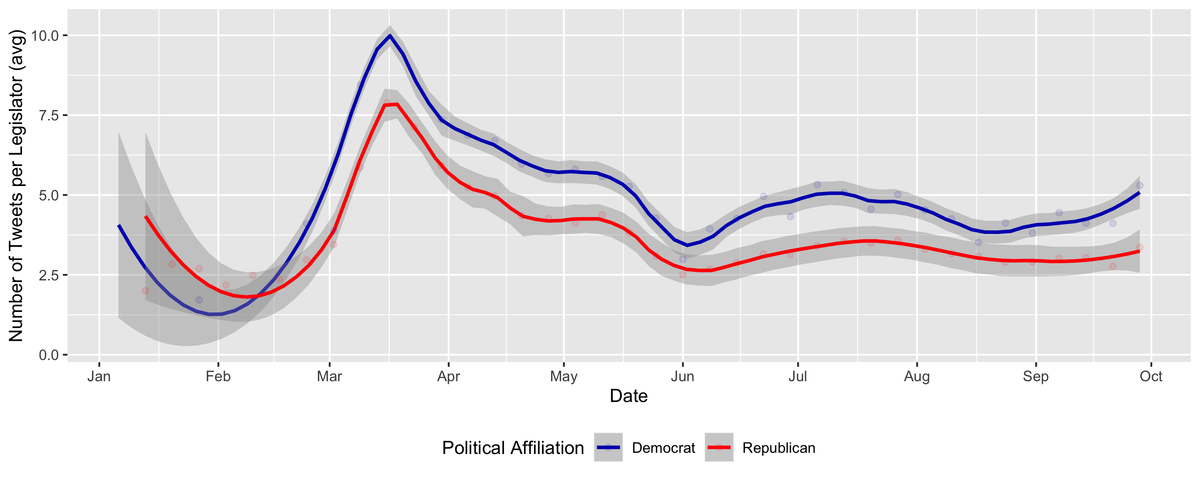

Supplement: Multimedia Appendix 1 [file jmir_v23i6e27300_app1.png]

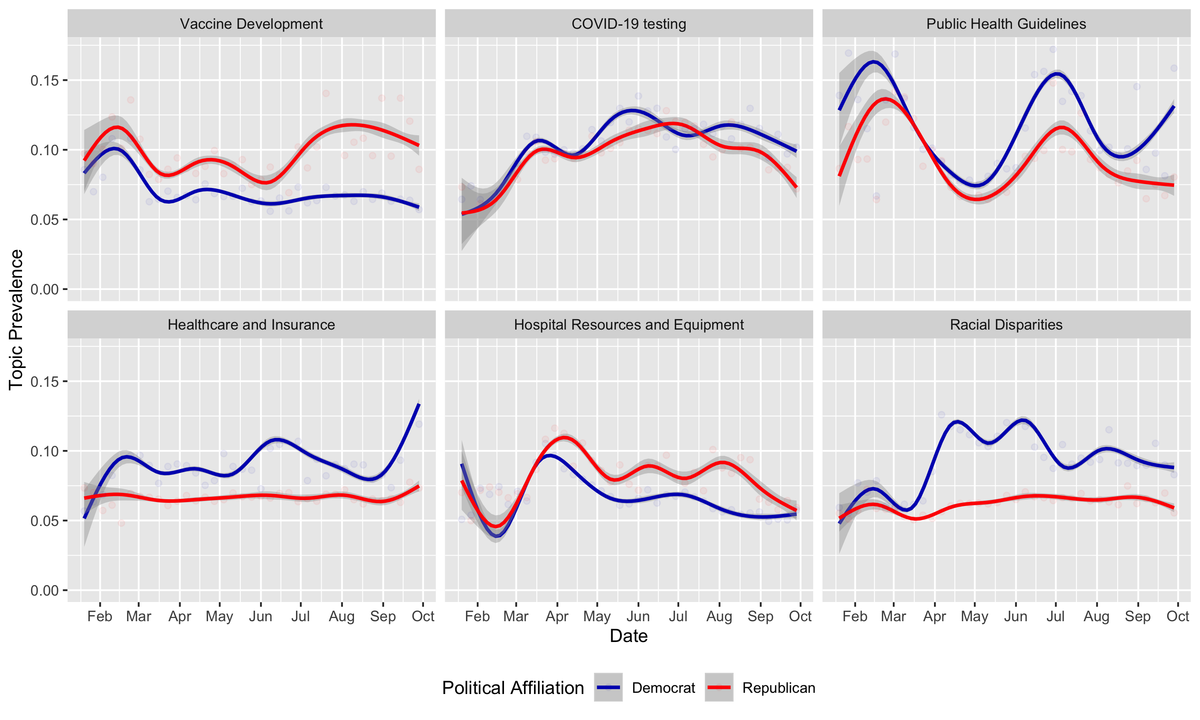

Supplement: Multimedia Appendix 2 [file jmir_v23i6e27300_app2.png]

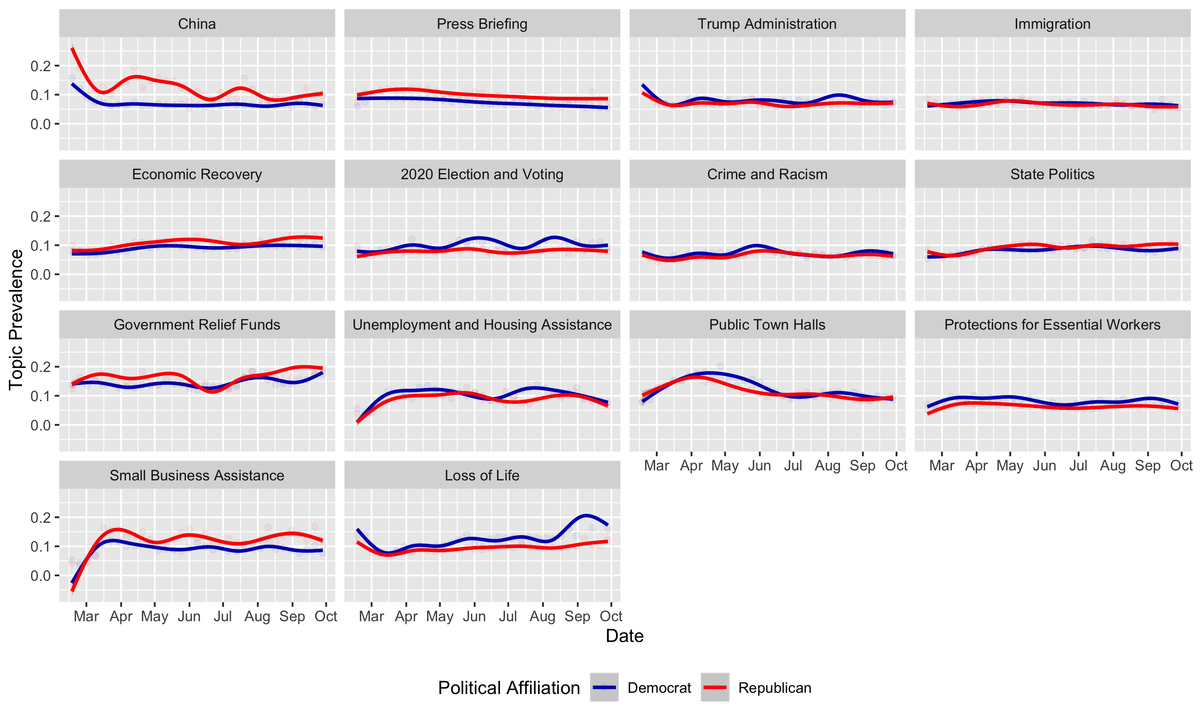

Supplement: Multimedia Appendix 3 [file jmir_v23i6e27300_app3.png]

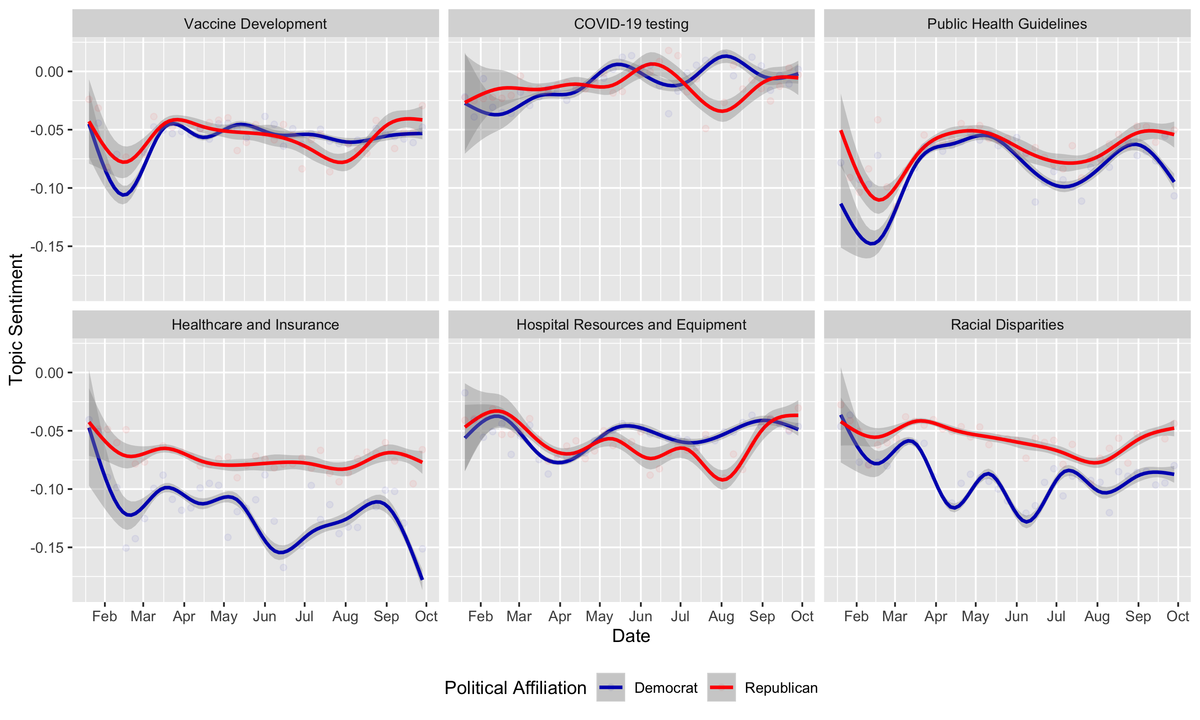

Supplement: Multimedia Appendix 5 [file jmir_v23i6e27300_app5.png]

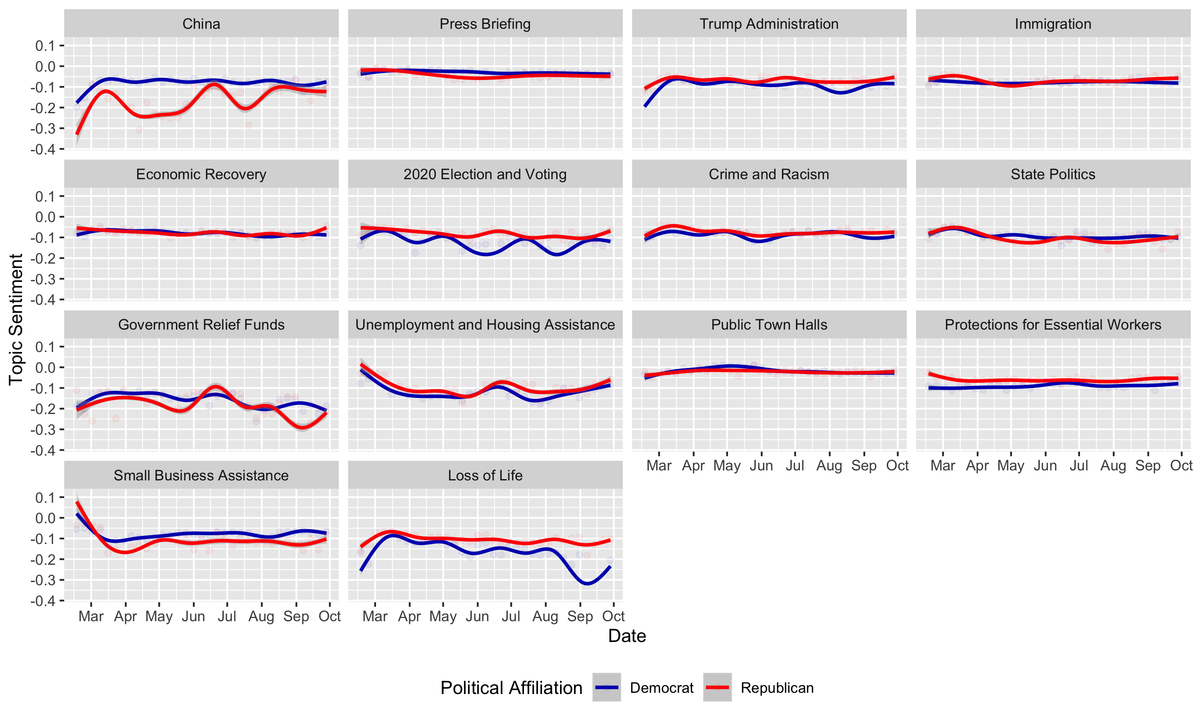

Supplement: Multimedia Appendix 6 [file jmir_v23i6e27300_app6.png]
