# Supplementary material for: Partisan Differences in Twitter Language Among US Legislators During the COVID-19 Pandemic: Cross-sectional Study
Source: J Med Internet Res. 2021 Jun 3;23(6):e27300. doi: 10.2196/27300 (PMC8176946; doi:10.2196/27300)
Supplement: Multimedia Appendix 4 [file jmir_v23i6e27300_app4.docx]

| Democrat | |
| --- | --- |
| **Top words in topic** | **Odds Ratio (95% CI)** |
| health, public, response, officials, experts, safety, cdc, dr, data, administration, top, science, government, threat, disease | 1.09 (1.05, 1.12) |
| health, call, mental, services, office, questions, hotline, contact, department, reach, phone, stress, email, concerns, issues | 1.07 (1.04, 1.1) |
| students, school, schools, education, child, children, kids, teachers, parents, learning, access, year, broadband, internet, online | 1.06 (1.03, 1.09) |
| i'm, work, working, support, continue, community, proud, hard, communities, ensure, challenges, fight, times, grateful, fighting | 1.06 (1.03, 1.09) |
| workers, front, care, lines, day, nurses, healthcare, working, responders, heroes, lives, fight, safe, fighting, doctors | 1.05 (1.01, 1.08) |
| day, year, happy, love, weekend, 2020, season, hope, football, sports, events, big, years, team, night | 1.02 (0.99, 1.05) |
| rep, senator, sen, #mapoli, dr, missouri, massachusetts, john, #moleg, mark, virginia, congressman, ceo | 1.02 (0.99, 1.05) |
| testing, test, tests, tracing, contact, plan, capacity, results, national, safely, strategy, reopen, increase, kits, widespread | 1.01 (0.98, 1.04) |
| Republican | |
| **Top words in topic** | **Odds Ratio (95% CI)** |
| information, resources, visit, website, updates, latest, check, info, find, update, page, resource, link, questions, updated | 1.12 (1.09, 1.16) |
| texas, county, #txlege, restaurants, local, texans, city, open, san, bars, abbott, close, mayor, reopen, houston | 1.11 (1.08, 1.15) |
| food, families, farmers, program, assistance, meals, free, producers, children, snap, impacted, insecurity, supply, hungry, agriculture | 1.11 (1.07, 1.14) |
| order, governor, due, announced, emergency, executive, march, 2020, gov, issued, april, closed, orders, extended, office | 1.1 (1.07, 1.14) |
| tax, money, payments, budget, pay, stimulus, taxes, billion, dollars, cuts, debt, cut, student, spending, financial | 1.1 (1.06, 1.13) |
| de, la, el, en, para, los, del, las, por, con, sobre, al, se, una, mas | 1.07 (1.04, 1.11) |
| cases, states, arizona, az, rate, reopening, york, numbers, weeks, number, colorado, surge, rates, spike, rise | 1.06 (1.03, 1.09) |
| positive, tested, symptoms, recovery, test, quarantine, days, negative, full, lady, staff, speedy, exposed, results, hope | 1.05 (1.02, 1.08) |
| house, session, senate, bill, legislature, legislative, special, #mnleg, committee, passed, budget, assembly, minnesota, bills, legislation | 1.05 (1.01, 1.08) |
| nursing, homes, home, care, residents, facilities, vulnerable, seniors, veterans, protect, long-term, older, risk, va, patients | 1.04 (1.01, 1.07) |
| virus, people, it's, don't, flu, risk, doesn't, science, disease, isn't, sense, dangerous, fear, deadly, common | 1.04 (1.01, 1.07) |
| cases, deaths, county, total, confirmed, positive, reported, number, case, data, update, numbers, today's, daily, statewide | 1.04 (1.01, 1.07) |
| #coronavirus, florida, read, desantis, #district13, #sd13, maine, #ksleg, kansas, #mepolitics, floridians | 1.03 (1, 1.06) |
| don't, good, you're, lot, i've, scams, there's, price, bad, things, thing, taking, they're, advantage, question | 1.02 (0.99, 1.05) |
| spread, stay, safe, virus, home, healthy, protect, prevent, stop, important, continue, slow, steps, lives, taking | 1.02 (0.99, 1.05) |
| cases, deaths, iowa, rate, days, tests, total, counties, 10, reported, 24, march, june, iowans, death | 1.01 (0.98, 1.04) |
| act, bill, relief, legislation, families, package, bipartisan, passed, house, support, congress, response, provide, senate, i'm | 1 (0.97, 1.03) |
